# Antibacterial and antifungal potentials of the solvents extracts from *Eryngium caeruleum*, *Notholirion thomsonianum* and *Allium consanguineum*

**DOI:** 10.1186/s12906-016-1465-6

**Published:** 2016-11-24

**Authors:** Abdul Sadiq, Sadiq Ahmad, Rahmat Ali, Fawad Ahmad, Sajjad Ahmad, Anwar Zeb, Muhammad Ayaz, Farhat Ullah, Abu Nasar Siddique

**Affiliations:** 1 Department of Pharmacy, University of Malakand, Chakdara, 18000 Dir (L), KPK Pakistan; 2 Department of Biotechnology, Bacha Khan University, Charsadda, 24420 KPK Pakistan

**Keywords:** *Notholirion thomsonianum*, *Allium consanguineum*, *Eryngium caeruleum*, Antibacterial, Antifungal, ZOI, MIC, MFC

## Abstract

**Background:**

Herbal medicines have long been used for various ailments in various societies and natural bioactive compounds are gaining more and more importance due to various factors. In this context, three plant species i.e., *Eryngium caeruleum*, *Notholirion thomsonianum* and *Allium consanguineum* have been aimed for the scientific verification of their purported traditional uses against various infectious diseases.

**Methods:**

In this study, three plants were assayed for antibacterial and antifungal potentials. The antibacterial investigations were performed via well diffusion method and nutrient broth dilution method. The bacterial strains used in the study were *Enterococcus faecalis*, *Proteus mirabilis*, *Escherichia coli*, *Salmonella typhi*, *Klebsiella pneumonia* and *Pseudomonas aeruginosa*. The antifungal potential was investigated by dilution method of Muller-Hinton agar media of the plants’ samples. The fungal strains used were *Aspergillis fumigatus*, *Aspergillis flavus* and *Aspergillis niger*. Ceftriaxone and nystatin were used as standard drugs in antibacterial and antifungal assays respectively.

**Results:**

Different fractions from *N. thomsonianum* were tested against five bacterial strains while the samples from *A. consanguineum* and *E. caeruleum* were tested against six bacterial strains. All the samples exhibited prominent antibacterial activity against the tested strains. Overall, chloroform and ethyl acetate fractions were found most potent among the three plants’ samples. *N. thomsonianum* excelled among the three plants in antibacterial activity. Similarly, in antifungal assay, *N. thomsonianum* exhibited strong antifungal activity against the fungal strains. The chloroform fraction displayed MFCs of 175.67 ± 5.20***, 29.33 ± 5.48*** and 63.00 ± 4.93*** μg/ml against *Aspergillus fumigatus*, *Aspergillus flavus* and *Aspergillus niger* respectively. The whole study demonstrates that all the three plant species were active against tested bacterial and fungal strains.

**Conclusion:**

It can be concluded from our findings that *N. thomsonianum*, *A. consanguineum* and *E. caeruleum* have broad antibacterial and antifungal potentials. In all of the plants’ samples, chloroform and ethyl acetate fractions were more active. Furthermore, being the potent samples, the chloroform and ethyl acetate fractions of these plants can be subjected to column chromatography for the isolation of more effective antimicrobial drugs.

## Background

Herbal therapies have long history for their use in various ailments. Being comparatively harmless, the natural products have attracted the focus of innovative researchers in the treatment of various challenging diseases [1, 2]. Microbial infections including tuberculosis, urinary tract infections (UTI’s), meningitis, dermatitis, otitis media caused by bacterial and fungal strains are among the challenging diseases for the scientific community [3–5]. The morbidity rate of diseases originated from bacteria and fungi is surpassing various other diseases [6, 7]. The use of antibacterial and antifungal drugs is a main approach among the therapeutic options to treat bacterial and fungal infections [8]. But still there are numerous factors which minimize the therapeutic outcome of the antibiotic therapy. Beside various factors, the microbial resistance has prominently diminished the efficacy of antibiotics and the microbial resistance is the major cause of failure to treat bacterial and fungal infections [9, 10]. Moreover, adverse drug reactions and hypersensitivity reactions associated with the use of various synthetic antimicrobial agents have decreased the interest of scientists to synthesize novel drugs having antimicrobial potentials. Therefore, the attention of researchers is mainly focused towards the natural compounds isolated from various plants [11–16]. Plants have been reported to possess antimicrobial potentials due to the presence of various secondary metabolites [17, 18]. The alkaloids and flavonoids have been reported to possess strong antimicrobial potentials against bacteria and fungi [19, 20]. Flavonoids like robinetin, myricetin, apigenin, rutin, Kuwanon C, mulberrofuran G, albanol B, kenusanone A and sophoraflavanone G isolated from various plants like *Morus alba* L., *Morus mongolica* Schneider, *Broussnetia papyrifera* (L.) Vent, *Sophora flavescens* Ait and *Echinosophora koreensis* Nakai have been reported to possess strong antibacterial potentials [19]. Similarly, alkaloids like sampangine and azafluorenone isolated from *Cananga odorata* and *Mitrephora diversifolia* respectively have also been reported to possess strong antifungal activities [21–24]. Varieties of plants belonging to different families have been scientifically verified for antimicrobial potentials. The scientific verification of a specific plant for the specific pharmacological activity is based on the traditional knowledge of species from the plant family. A specific biological potential of specific plant can be heralded by the ethnobotany and ethnomedicine [25].

*Eryngium caeruleum* belongs to the family Apiaceae. Several species of this genus have been reported to possess antimicrobial activities [26]. Similarly, *Notholirion thomsonianum* belongs to the family Liliaceae. This plant has been used ethnomedicinally for the treatment of various infectious diseases especially intestinal [27]. Likewise, *Allium consanguineum* belongs to the family Amaryllidaceae. A wide variety of species of Allium genus have been reported to possess notable antimicrobial properties in which the onion and garlic are the prominent candidates [28–30]. The selection of these plants species for antimicrobial studies was made on the basis of their traditional uses as well as their genera and family background. Similarly, the rhizomes of *N. thomsonianum* and *A. consanguineum* were exploited for extraction due to ethnomedicinal use of rhizome. Secondly, the rhizomes and underground part of multiple plants of the families of these plants have been reported to be rich sources of numerous bioactive compounds i.e., *Allium sativum* and *Allium cepa* [31]. Likewise, the aerial parts of several species of Eryngium are reportedly good source of secondary metabolites and possess pharmacological potentials [32]. Therefore, the aerial parts of *E. caeruleum* were employed for extraction. Therefore, the current study was aimed to evaluate the antibacterial and antifungal potentials of *Eryngium caeruleum, Notholirion thomsonianum* and *Allium consanguineum* and scientifically validate its folkloric uses.

## Methods

### Plant collection and extraction

The plants used in this research, i.e. *E. caeruleum*, *N. thomsonianum* and *A. consanguineum* were collected from Malakand division, KPK, Pakistan, located at Latitude: 34.5030° N and Longitude: 71.9046° E and authenticated by Dr. Nasrullah at Department of Botany, University of Malakand, Pakistan. Sample of each plant is submitted/stored in the herbarium of University of Malakand, Pakistan. The voucher numbers given by the herbarium officials are H.UOM.BG.109 (*E. caeruleum*) H.UOM.BG.106 (*N. thomsonianum*) and H.UOM.BG.158 (*A. consanguineum*). The rhizomes of the *N. thomsonianum* and *A. consanguineum* were isolated from the plants weighing approximately 3 kg each, divided into small pieces and shade dried for 2 to 3 weeks. Likewise, the aerial parts of *E. caeruleum* were collected and dried in the shade. After drying, each plant materials were cut using a cutter mill into a powder and macerated in 80% methanol for 2 weeks. After individual soaking these plant samples were filtered using Whattman filter paper (Whatman no. 1). The filtrates were evaporated using rotary evaporator (Heidolph Laborota 4000, Schwabach, Germany) at 40°C under reduced pressure [33, 34]. Semi solid masses of methanolic extracts of *E. caeruleum*, *N. thomsonianum* and *A. consanguineum* were obtained weighing approximately 400 g each.

### Fractionation

The successive solvent-solvent extraction procedure was followed for the fractionation of these plants samples. The crude methanolic extracts of *E. caeruleum*, *N. thomsonianum* and *A. consanguineum* (300 g each) were suspended separately in 500 ml of distilled water in separating funnels and diluted with 500 ml of *n*-hexane. After vigorously shaking, all the three extracts were allowed to separate into two distinct layers. The upper *n*-hexane layer was collected and the same procedure was repeated until colorless *n*-hexane layer was obtained. After the collection of *n*-hexane fraction of each plant, it was fractionated with other solvents with increasing polarity i.e., chloroform, ethyl acetate and at last the aqueous fraction was obtained. The weights obtained for *n*-hexane, chloroform, ethyl acetate and aqueous fractions of *E. caeruleum* were 62 g (20. 66%), 37 g (12.33%), 59 g (19.66%) and 110 g (36.66%) respectively. Similarly, the weights obtained for *n*-hexane, chloroform, ethyl acetate and aqueous fractions of *N. thomsonianum* were 69 g (23%), 45 g (15%), 51 g (17%) and 81 g (27%) respectively. Likewise, the weights obtained for *n*-hexane, chloroform, ethyl acetate and aqueous fractions of *A. consanguineum* were 45 g (15%), 37 g (12.33%), 64 g (21.33%) and 96 g (32%) respectively [35–38].

### Bacterial and fungal strains

Antibacterial potential of plants’ samples were investigated against *Enterococcus faecalis* (stools), *Esherichia coli* (UTI), *Proteus mirabilis* (UTI), *Pseudomonas aeruginosa* (Burn patient skin), *Klebsiella pneumoniae* (UTI), *Salmonella typhi* (Stools). All bacterial and fungal strains were kindly donated by Department of Microbiology, Quaid-e-Azam University, Islamabad, Pakistan. These were identified by various biochemical tests and were kept at 4°C in agar slants in freeze-dried condition until later use [39]. The fungicidal activity was determined against *Aspergillus fumigates* (patient’s sputum)*, Aspergillus niger* (grapes) and *Aspergillus flavus* (soil) were used*.*

### Preparation and standardization of bacterial and fungal strains

The cultures of bacterial strains were prepared by incubating at 37°C for 24 h. The suspensions of bacterial strains with cell density of 1 × 10^8^ CFU/ml were prepare by comparing with McFarland standard No 2 and was later on diluted to a cell density of 1 × 10^6^ CFU/ml through double beam UV-visible spectrophotometer (Thermo electron corporation USA) at 625 nm. Fungal strains were grown at 25°C and suspensions corresponding to 2.5 × 104 cells ml − 1 were prepared by dilution in normal saline. Standardization of fungal strains were done using microscopic enumeration with a cell-counting hematocytometer and optical density method as previously reported [40]. Fungal strains were identified according to principles and procedures of clinical laboratory standard institute (CLSI) for the detection of fungi in clinical specimens [41].

### Antibacterial assay

The well-diffusion method was used for the evaluation of antibacterial activity of various samples of the three plants [42, 43]. Nutrient agar plates were prepared, properly labeled and inoculated with the test organisms under laminar flow hood with aseptic conditions. Wells having diameter of 5 mm were made in the agar plate using a sterilized cork borer. Samples of various extracts of plants were prepared having concentration of 10 mg/ml. Samples of each plant having volume of 100 μl were transferred into the respective wells of petri dishes using micropipette. In each petri plate, four wells at the sides and one well in the center were made.

In the center one, ceftriaxone (1 mg/ml) was added which served as positive control while in the rest of wells, the plant samples were added. The petri dishes were kept at 37°C in BOD incubator for 24 h. After incubation, the zone of inhibition of each sample was measured in mm. All the samples were run in triplicate and the data obtained was expressed as mean ± SEM.

### Determination of MICs

For the determination of minimum inhibitory concentration (MICs), the broth dilution method was employed. Briefly, stock solution having the concentration of 50 mg/ml was prepared in sterile distilled water. Various dilutions were prepared from the stock solution ranging from 0.125 to 10 mg/ml. Nutrient broth media was prepared in sterile water and sterilized in autoclave. The media prepared was inoculated with various strains in separate conical flasks. A few ml of inoculated media was transferred aseptically into properly labeled test tubes under laminar flow hood and the test samples were added to them. The test tubes were incubated at 37°C for 24 h. After incubation, the test tubes were observed for turbidity which is directly related to the growth of bacterial strains. Similarly, the MICs of each plant’s sample were recorded as the maximum concentration at which no turbidity was observed [44–46].

### Determination of MFCs

Antifungal activity was carried out for the plants’ samples against *A. fumigatus, A. niger* and *A. flavus.* The plants’ samples of various dilutions (62.5 μg/ml to 10 mg/ml) were prepared in DMSO. Fungal media, i.e. muller hinton agar media was prepared in sterile water and autoclaved. A few ml of the prepared media was transferred to the labeled test tubes and 1 ml of plants’ sample was added to each of them. The test tubes were inoculated with test strains and incubated in BOD incubator at 25°C for 8 days. After incubation, the test tubes were observed for fungal growth. The minimum fungicidal concentrations (MFCs) were recorded as the maximum concentration of the sample at which no fungal growth was observed in test tubes. All the procedure was performed in triplicate and the nystatin was employed as positive control [47, 48].

### Statistical analysis

Two-way ANOVA followed by Bonferroni’s multiple comparison test was applied for the comparison of positive control with the test groups. *P* values less than or equal to 0.05 were considered statistically significant. The standard error of mean (SEM) were calculated at 95% confidence intervals.

## Results

### Antibacterial assays

#### Zone of inhibitions (ZOIs) determination

The well diffusion method was used for the three plants against various bacterial strains. The crude extracts and sub-fractions were analyzed for their antibacterial effect by the determination of their inhibitory zones against each strain. Among the three plants, *N. thomsonianum* exhibited the highest inhibitory zones against the tested strains. The Nt.Cf exhibited 31.67 ± 0.67, 29.33 ± 0.88, 26.33 ± 0.33, 25.67 ± 0.67 and 29.33 ± 0.88 mm zone of inhibitions (ZOI) against *P. mirabilis, E. coli, S. typhi, K. pneumonia* and *P. aeruginosa* respectively as shown in Table 1. The antibacterial effect of Nt.Cf was almost comparable to the effects of the positive control. In the remaining fractions of *N. thomsonianum*, Nt.EtAc and Nt.Cr also demonstrated notable antibacterial potentials. The least activity is attributed to the Nt.Hex fraction.

**Table 1:** Zone of inhibitions of the solvents fractions in millimeter from three plants against various bacterial strains

Plant	Samples	*Enterococcus faecalis*	*Proteus mirabilis*	*Escherichia coli*	*Salmonella typhi*	*Klebsiella pneumonia*	*Pseudomonas aeruginosa*
*Eryngium caeruleum*	Ec.Cr	11.16 ± 0.60***	12.16 ± 0.44***	7.83 ± 0.72***	17.66 ± 0.88***	15.83 ± 1.01***	7.16 ± 0.60***
	Ec.Hex	11.66 ± 0.88***	13.16 ± 0.44***	6.83 ± 1.01***	16.16 ± 0.72***	12.83 ± 0.92***	9.16 ± 0.44***
	Ec.Cf	14.16 ± 0.72**	17.33 ± 0.44***	7.16 ± 0.60***	15.16 ± 0.44***	18.33 ± 0.92***	20.16 ± 0.44***
	Ec.EtAc	16.16 ± 0.72*	13.16 ± 0.60***	7.66 ± 0.88***	18.33 ± 0.72***	20.66 ± 0.88***	11.50 ± 1.04***
	Ec.Aq	13.50 ± 0.86**	12.83 ± 0.92***	8.16 ± 0.72***	13.16 ± 0.44***	17.16 ± 0.44***	12.83 ± 0.92***
	Ceftr	21.30 ± 0.41	39.67 ± 0.33	32.67 ± 0.88	36.67 ± 0.67	32.67 ± 0.33	39.33 ± 0.33
*Notholirion thomsonianum*	Nt.Cr	Nd	20.33 ± 0.33***	29.00 ± 0.58*	25.33 ± 0.33***	16.67 ± 0.33***	22.00 ± 0.58***
	Nt.Hex	Nd	17.33 ± 0.33***	17.67 ± 0.67***	-	16.33 ± 0.33***	07.00 ± 0.58***
	Nt.Cf	Nd	31.67 ± 0.67***	29.33 ± 0.88*	26.33 ± 0.33***	25.67 ± 0.67***	29.33 ± 0.88***
	Nt.EtAc	Nd	26.67 ± 0.33***	21.00 ± 0.58***	21.67 ± 0.67***	14.33 ± 0.88***	09.33 ± 0.88***
	Nt.Aq	Nd	29.33 ± 0.33***	-	16.67 ± 0.33***	27.67 ± 0.67**	26.67 ± 0.33***
	Ceftr	21.3 ± 0.41	39.67 ± 0.33	32.67 ± 0.88	36.67 ± 0.67	32.67 ± 0.33	39.33 ± 0.33
*Allium consanguineum*	Ac.Cr	9.66 ± 0.88***	13.33 ± 1.20***	6.83 ± 0.60***	19.33 ± 0.88***	17.66 ± 1.45***	6.33 ± 0.88***
	Ac.Hex	11.33 ± 0.66***	12.66 ± 0.88***	5.83 ± 0.60***	16.30 ± 0.88***	13.66 ± 0.88***	8.83 ± 0.92***
	Ac.Cf	15.50 ± 0.86*	16.16 ± 0.72***	7.00 ± 0.50***	14.50 ± 0.86***	19.16 ± 0.44***	21.36 ± 0.57***
	Ac.EtAc	15.50 ± 0.76*	12.66 ± 0.88***	6.83 ± 0.92***	19.16 ± 0.44***	20.83 ± 0.44***	10.33 ± 0.60***
	Ac.Aq	14.50 ± 0.76**	13.50 ± 1.04***	7.10 ± 0.58***	12.50 ± 0.28***	16.33 ± 0.88***	9.83 ± 0.44***
	Ceftr	21.3 ± 0.41	39.67 ± 0.33	32.67 ± 0.88	36.67 ± 0.67	32.67 ± 0.33	39.33 ± 0.33

Values significantly different as compared to standard drug i.e. ***: *p* < 0.001, **: *p* < 0.01 and *: *p* < 0.05

The *A. consanguineum* also showed notable inhibitory zones against the tested strains. Among different samples of *A. consanguineum*, the Ac.EtAc and Ac.Cf displayed considerable ZOIs in comparision to the positive control. The Ac.EtAc demonstrated 15.50 ± 0.76, 12.66 ± 0.88, 6.83 ± 0.92, 19.16 ± 0.44, 20.83 ± 0.44 and 10.33 ± 0.60 mm ZOIs against *E. faecalis, P. mirabilis, E. coli, S. typhi, K. pneumonia* and *P. aeruginosa* respectively (Table 1). Moreover, almost all the solvent fractions of *E. caeruleum* were active against all the strains but the antibacterial effect of this plant was relatively low compared to the other two plants. However, the chloroform and ethyl acetate fractions were still observed to have dominant antibacterial activity amongst other fractions of *E. caeruleum*.

#### Minimum inhibitory concentrations (MICs) determination

The minimum concentration of each sample of *N. thomsonianum, A. consanguineum* and *E. caeruleum* was evaluated using agar dilution method in which all the samples exhibited specific MIC values. The MIC values of the three plants are summarized in Table 2. Among the test samples, the chloroform fractions (Nt.Cf, Ac.Cf, Ec.Cf) showed the least MIC values against all the test strains. It means that the chloroform fractions of the three plants were the most active among the rest of the samples. The chloroform fraction of *N. thomsonianum* exhibited MIC values of 39.67 ± 4.05, 280.00 ± 3.00, 47.33 ± 3.52, 73.33 ± 2.84 and 63.33 ± 2.84 μg/ml against *P. mirabilis*, *E. coli*, *S. typhi*, *K. pneumonia* and *P. aeruginosa* respectively. The chloroform fraction of *A. consanguneum* was observed with 104.76 ± 6.21, 208.64 ± 4.86, 83.34 ± 4.21, 416.65 ± 4.83, 166.53 ± 4.26 and 208.65 ± 4.76 μg/ml against *E. faecalis*, *P. mirabilis*, *E. coli*, *S. typhi*, *K. pneumonia* and *P. aeruginosa* respectively. As obvious from our results in Tables 1 (ZOIs) and Table 2 (MICs) that *E. caeruleum* is comparatively low potent plant in antibacterial activity. However, among all the solvent fractions of *E. caeruleum*, chloroform and ethyl acetate fractions were observed with minimum inhibitory zones confirming the antibacterial possibility of this plant.

**Table 2:** Minimum inhibitory concentrations of the solvents fractions from three plants against various bacterial strains in μg/ml

Plant	Samples	*Enterococcus faecalis*	*Proteu mirabillis*	*Escherichia coli*	*Salmonella typhi*	*Klebsiella pneumonia*	*Pseudomonas aeruginosa*
*Eryngium caeruleum*	Ec.Cr	309.43 ± 2.08***	380.34 ± 4.17***	326.33 ± 7.12***	653.74 ± 4.48***	774.65 ± 3.28***	823.64 ± 6.37***
	Ec.Hex	386.70 ± 3.20***	583.74 ± 2.20***	316.64 ± 0.31***	700.35 ± 5.570***	614.64 ± 5.575***	526.43 ± 5.27***
	Ec.Cf	420.45 ± 4.21***	416.73 ± 6.83***	540.40 ± 3.30***	686.35 ± 3.33***	273.74 ± 4.371***	456.64 ± 2.47***
	Ec.EtAc	433.50 ± 6.29***	470.32 ± 4.25***	486.74 ± 6.52***	556.32 ± 3.66***	393.75 ± 4.104***	373.29 ± 6.371***
	Ec.Aq	523.72 ± 5.28***	703.64 ± 4.46***	846.35 ± 3.63***	983.64 ± 6.440***	116.36 ± 7.30***	873.36 ± 4.371***
	Ceftr	2.03 ± 0.053	3.867 ± 0.384	2.367 ± 0.120	1.67 ± 0.167	0.503 ± 0.317	1.67 ± 0.120
*Notholirion thomsonianum*	Nt.Cr	nd	87.67 ± 1.20***	58.67 ± 2.18***	179.67 ± 1.76***	933.33 ± 1.45***	501.33 ± 1.85***
	Nt.Hex	nd	176.67 ± 2.02***	255.33 ± 2.60***	-	1132.00 ± 4.35***	913.67 ± 3.48***
	Nt.Cf	nd	39.67 ± 4.05***	280.00 ± 3.00***	47.33 ± 3.52***	73.33 ± 2.84***	63.33 ± 2.84***
	Nt.EtAc	nd	768.33 ± 1.85***	404.67 ± 2.72***	534.33 ± 2.40***	504.00 ± 3.51***	388.67 ± 1.76***
	Nt.Aq	nd	803.33 ± 3.33***	-	723.33 ± 3.93***	512.67 ± 3.84***	633.67 ± 3.71***
	Ceftr	2.03 ± 0.053	3.867 ± 0.384	2.367 ± 0.120	1.67 ± 0.167	0.503 ± 0.317	1.67 ± 0.120
*Allium consanguineum*	Ac.Cr	333.45 ± 4.833***	666.86 ± 6.66***	208.83 ± 4.16***	583.54 ± 2.20***	833.43 ± 1.66***	666.29 ± 7.66***
	Ac.Hex	416.24 ± 6.83***	416.22 ± 8.38***	333.73 ± 8.33***	833.75 ± 3.66***	500.00 ± 0.00***	166.45 ± 4.16***
	Ac.Cf	104.76 ± 6.211***	208.64 ± 4.86***	83.34 ± 4.21***	416.65 ± 4.83***	166.53 ± 4.26***	208.65 ± 4.76***
	Ac.EtAc	208.64 ± 3.48***	166.54 ± 5.41***	416.65 ± 5.83***	333.54 ± 6.83***	208.36 ± 3.41***	25.00 ± 0.00***
	Ac.Aq	761.64 ± 3.89***	684.53 ± 7.66***	1333.54 ± 4.83***	833.63 ± 5.32***	833.64 ± 3.66***	666.53 ± 1.66***
	Ceftr	203 ± 0.053	3.867 ± 0.384	2.367 ± 0.120	1.67 ± 0.167	0.503 ± 0.317	1.67 ± 0.120

Values significantly different as compared to standard drug i.e. ***: *p* < 0.001, **: *p* < 0.01 and *: *p* < 0.05

#### Minimum fungicidal concentrations (MFCs)

The antifungal effect of each sample was studied and the minimum fungicidal concentration was determined which revealed the least MFC values (high potential) of *N. thomsonianum*. Among the different fractions of *N. thomsonianum*, Nt.Cf exhibited the highest antifungal potential with MFC values of 175.67 ± 5.20, 29.33 ± 5.48 and 63.00 ± 4.93 μg/ml against *A. fumigatus, A. flavus* and *A. niger* respectively as shown in Table 3. All the samples of the three plants were found active against all the three fungal strains. Among the samples of *A. consanguneum*, chloroform and *n*-hexane fractions were found to be most active exhibiting overwhelming MFC values compared to other fractions. Similarly, the *E. caeruleum* was also found quite active against all the three fungal strains. The most active fraction of this plant was found to be Ec.Cf, which exhibited MFC values of 350.23 ± 2.28, 233.45 ± 6.44 and 250.64 ± 4.76 μg/ml against *A. fumigatus, A. flavus* and *A. niger* respectively.

**Table 3:** Minimum fungicidal concentrations of the solvents fractions from three plants expressed as μg/ml

Plant	Fungal strains	Methanolic extract	*n*-Hexane fraction	Chloroform fraction	Ethylacetate fraction	Aqueous fraction	Nystatin
*Eryngium caeruleum*	1	321.43 ± 2.43***	433.33 ± 4.33***	350.23 ± 2.28***	450.75 ± 3.76***	616.25 ± 2.60***	13.67 ± 3.75
	2	416.76 ± 5.44***	333.96 ± 2.60***	233.45 ± 6.44***	450.43 ± 5.57***	516.63 ± 5.72***	9.33 ± 2.60
	3	333.92 ± 3.88***	366.73 ± 5.92***	250.64 ± 4.76***	450.75 ± 2.86***	616.75 ± 3.88***	31.67 ± 5.78
*Notholirion thomsonianum*	1	254.67 ± 2.72***	412.67 ± 4.33***	175.67 ± 5.20***	225.33 ± 3.93***	301.67 ± 4.40***	13.67 ± 3.75
	2	73.67 ± 4.40***	585.33 ± 3.93***	29.33 ± 5.48***	213.67 ± 4.25***	514.67 ± 4.33***	9.33 ± 2.60
	3	116.67 ± 3.84***	232.67 ± 4.40***	63.00 ± 4.93***	309.33 ± 5.48***	382.67 ± 4.40***	31.67 ± 5.78
*Allium consanguineum*	1	391.52 ± 1.90***	364.56 ± 2.19***	329.65 ± 3.16***	372.43 ± 3.57***	211.00 ± 3.63***	13.67 ± 3.75
	2	713.76 ± 2.91***	380.74 ± 4.15***	790.00 ± 3.17***	403.24 ± 6.23***	513.74 ± 2.24***	9.33 ± 2.60
	3	310.78 ± 2.63***	326.83 ± 8.12***	280.83 ± 5.65***	502.65 ± 3.15***	540.64 ± 5.20***	31.67 ± 5.78

Values significantly different as compared to standard drug i.e. ***: *p* < 0.001, **: *p* < 0.01 and *: *p* < 0.05

Key: 1 = *Aspergillus fumigatus*; 2 = *Aspergillus flavus*; 3 = *Aspergillus niger*

## Discussion

Microbes are considered as the mainstay and origin of multiple diseases. Microbial infiltration into the body tissues and blood lead to various diseases some of which are difficult to treat and extremely lethal [49, 50]. The etiology of various humans’ infections may be related to bacteria, virus, fungi and protozoa. The bacterial infections like upper respiratory tract infection, lower respiratory tract infection, tuberculosis, pneumonia, bacillary dysentery etc. are getting more and more attention due to the increasing morbidity and mortality due to these infections [51, 52]. Similarly, most of the fungi have the ability to cause infection without getting entered into the blood stream or mucosa. Such type of fungal infections involve disruption of dermal layer and are contagious [53]. Moreover, the fungi may cause a lot of systemic infections as well [54]. To avoid various health complications, a wide variety of antimicrobial drugs are used which avoid and alleviates the symptoms caused by microbes. The antimicrobial agents may interrupt the synthesis of metabolites which are necessary for the basic integrity of microbes including bacteria and fungi [55, 56]. However, the main problem with the use of antimicrobial and other drugs is the hazardous and toxic effects associated with their use [57, 58]. Secondly, many microbes develop resistance to a specific group of drugs and they become less susceptible to that specific drug [55]. Fortunately, nature is kind enough to provide remedy for almost every disease [59]. The natural bioactive compounds being biodegradable and hydrolytic have been reported to possess comparatively low toxicity and high efficacy [60]. Investigators are in continuous search for the exploration of novel sources of more effective compounds for the treatment of challenging infectious diseases [61–63]. As previously reported, plants possess antibacterial secondary metabolites which are getting more and more importance due to their negligible toxicity and adverse effects [64, 65]. The aim of the current study is to have a step towards the achievement of novel natural antimicrobial agents. Results of the current study revealed that *N. thomsonianum* possess strong antibacterial and antifungal results. The MIC values of positive control and chloroform fraction were going considerably parallel i.e., if we cursorily observe the Table 2, we can figure out the antibacterial potential in terms of MIC (39.67, 280.00, 47.33, 73.33, 63.33 μg/ml) of chloroform fraction and that of positive control (2.03, 3.867, 2.367, 1.67, 0.503, 1.67 μg/ml), which are relatively comparable. The MICs of chloroform fraction has been figured out to be smaller than 100 μg/ml against majority of the test strains, which reveals the significance of this fraction. This significance is in the terms of therapeutic applications against various bacterial infections. As variety of antimicrobial natural compounds and antibiotics have their MIC values lower than 100 μg/ml, which are effective against various bacterial infections and are considered as therapeutically as well as statistically important data [66, 67]. Similarly, the antifungal activity of chloroform fraction is also dominant. It was also observed that the chloroform fraction was the most active one against bacterial and fungal strains. Against *A. niger* and *A. flavus* the chloroform fraction demonstrated MFC values of 63.00 ± 4.93 and 29.33 ± 5.48 μg/ml respectively, which are less than 100 μg/ml, which can be considered to be significant in the context of therapy of various diseases [68]. The results obtained in the current study can be correlated with the previous publications. Other species of Allium also possess effective potential against microbes i.e., *Allium sativum* L. (garlic), *Allium ampeloprasum* L. (elephant garlic) and *Allium cepa* have been reported to possess strong antibacterial and antifungal activities [69]. Important antibacterial compound i.e., allicin has also been isolated from *Allium sativum* which has been reported to be active against various pathogenic bacterial strains [70]. In the same way, numerous species of Eryngium have been reported to possess antimicrobial potential. *Eryngium palmatum* one of medicinally important specie of Eryngium has been verified to be active against microbes [71]. Similarly, the Eryngium genus has also been reported to inhibit the methicillin‐resistant *Staphylococcus aureus* strains significantly [72]. The results of the previous reports goes parallel with the results of our current investigational study which indicate that like other species of Allium and Eryngium the *E. caeruleum, A. consanguineum* and *N. thomsonianum* are also prominent candidates of these genera. The study reveals that among the three plants, *N. thomsonianum* was rich in antimicrobial agents and secondly it may also be implied that the chloroform fraction was rich in such secondary metabolites which confer antimicrobial potential to this plant.

## Conclusion

Based on our current investigations it can be concluded that *N. thomsonianum, A. consanguineum* and *E. caeruleum* possess considerably sufficient antibacterial and antifungal potentials. It may also be concluded that antimicrobial potential of various samples of these plants might be due to wide variety of compounds present in these plants. Moreover, we observed that specifically the chloroform and upto some extent the ethyl acetate fractions of these plant contain considerably high activity. These two fractions (in each plant) are potent targets to be subjected to bio-guided isolation and exploration of novel natural antimicrobials.

## Abbreviations

Ac.Aq: Aqueous fraction of *A. consanguineum*
Ac.Cf: Chloroform fraction of *A. consanguineum*
Ac.Cr: Methanolic extract of *A. consanguineum*
Ac.EtAc: Ethyl acetate fraction of *A. consanguineum*
Ac.Hex: n-Hexane fraction of *A. consanguineum*
CLSI: Clinical laboratory standard institute
DMSO: Dimethyl sulfoxide
Ec.Aq: Aqueous fraction of *E. caeruleum*
Ec.Cf: Chloroform fraction of *E. caeruleum*
Ec.Cr: Methanolic extract of *E. caeruleum*
Ec.EtAc: Ethyl acetate fraction of *E. caeruleum*
Ec.Hex: n-Hexane fraction of *E. caeruleum*
MFC: Minimum fungicidal concentration
MIC: Minimum inhibitory concentration
Nt.Aq: Aqueous fraction of *N. thomsonianum*
Nt.Cf: Chloroform fraction of *N. thomsonianum*
Nt.Cr: Methanolic extract of *N. thomsonianum*
Nt.EtAc: Ethyl acetate fraction of *N. thomsonianum*
Nt.Hex: n-Hexane fraction of *N. thomsonianum*
UTI: Urinary tract infection
ZOI: Zone of inhibition
